# Computer Aided Patterning Design for Self-Assembled Microsphere Lithography (SA-MSL)

**DOI:** 10.1038/s41598-019-48881-z

**Published:** 2019-09-06

**Authors:** Rhiannon Lees, Michael D. Cooke, Claudio Balocco, Andrew Gallant

**Affiliations:** Department of Engineering, Durham University, Durham, DH1 3LE United Kingdom

**Keywords:** Surface patterning, Computational methods, Surface patterning, Surface patterning

## Abstract

In this paper, we use a finite difference time domain solver to simulate the near field optical properties of self-assembled microsphere arrays when exposed to an incoherent light source. Such arrays are typically used for microsphere lithography where each sphere acts as a ball lens, focusing ultraviolet light into an underlying photoresist layer. It is well known that arrays of circular features can be patterned using this technique. However, here, our simulations show that additional nanometer scale features can be introduced to the pattern by optimising the sphere dimensions and exposure conditions. These features are shown to arise from the contact points between the microspheres which produce paths for light leakage. For hexagonally close packed arrays, the six points of contact lead to star shapes in the photoresist. These star shapes have subfeature sizes comparable to the current achievable resolution of low-cost fabrication techniques.

## Introduction

Self-assembled microsphere lithography (SA-MSL) is a cost effective, fast, highly ordered, repeatable, and innovative method of microarray fabrication, the origins of which lie in the work of Van Duyne’s group^1^. A colloidal crystal mask (CCM) is used instead of a conventional mask which is applied directly onto the surface of the substrate. CCM’s can be formed by gravity sedimentation^2^, electrophoretic deposition^3^, solvent evaporation^4^, the Langmuir-Blodgett technique^5^, the air–water interfacial floating method^6^ and spin coating^7^. We chose spin coating due to the fact it is relatively cheap, fast and a common cost-effective equipment setup found in many laboratories.

Thorough and accurate simulations of incident illumination on the physical setup have been utilised to explore the effects and characteristics of the light within the photoresist. This enables the exploitation of the inherent phenomena of nanosphere lithography (NSL) without the use of unconventional, less cost-effective equipment. The resultant array of 2D 6-point microstars has nano-dimensional sub-features which can be produced to a length scale of the order of 100 nm. This is a comparable resolution to other nanofabrication techniques for example star arrays formed using electron beam lithography^8^. The stars produced in ref.^8^ are down to 1 *μ*m which has been reduced in this paper (the 2 *μ*m spheres produce a star of 800 nm). Projection photolithography is a method of NSL, forming complex designs by projecting an image through the microspheres forming an array of this image via the array of microsphere lenses. However, this method requires complex equipment and is limited to large microsphere sizes^9^. In this paper, a cost-effective method of producing star shapes by exploiting the inherent properties of the microspheres within the resist is demonstrated. The unique benefits of this work lies within the fabrication techniques utilising the complex optical response of the microspheres to produce the star shape, coupled with the simplicity and cheapness of the technique to produce potentially large areas of these features.

The microstar arrays could be utilised as an array itself or could be lifted off to be used as individual star shaped particles (microstars). NSL is already used for plasmonic enhancement by using deposition and lift off to form triangular shapes between the spheres^10^. Using NSL to produce star shaped arrays for plasmonic enhancement could provide more angular stability than its triangular counterpart. Reference^11^ uses NSL to produce micro rings which can be used for resonance; any periodic metallic shape has a resonance and this method is a cheap and simplistic way of producing an array of stars which are one of the more complex shapes, therefore they could show promise in this area. The unique shape of the microparticles produced here is expected to have a particularly useful topology; the points of the star are of particular interest as they lend themselves to increasing the likelihood of tunnelling within composite materials^12^. Whilst many applications already exist that utilise the topology of 3D star microparticles, 2D (flat) versions could have their own unique benefits in terms of reducing physical space. An important field which could benefit from these advancements is bioengineering in both biosensors and drug delivery. Both the arrays and the microstars could be of use in the development in label-free impedance biosensors^13^ and electrochemical sensors^14^. Reference^15^ stresses the importance of microparticle shape with respect to their uses for drug delivery. Currently a bottleneck in terms of development in this field is the production of easily fabricated alternate shapes. Therefore, advances such as those reported in this paper are critical to the future development of the microparticle-based drug delivery field.

## Results and Discussion

### Basic microsphere lithography theory

**Figure 1:**
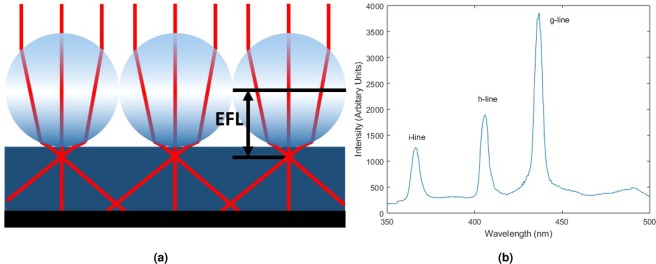
(**a**) Figure displaying the use of microspheres as lenses for lithography. The concentration of illumination and thus exposure creates the pattern within the resist. (**b**) Plot of mercury lamp spectrum using an EVG620 and a glass plate as the mask showing g, h and i-lines measured using an Ocean Optics USB2000. These three peaks measured in the spectrum are used to statistically model the incoherent light source in the simulations.

In microsphere lithography, a suspension of polystyrene microspheres is spin coated on top of a photoresist layer. Under certain spin coating conditions, these spheres can self-assemble into close packed arrays. When the array is top illuminated by a light source (e.g. from a UV mask aligner), as shown in Fig. 1a, each microsphere acts as a ball lens which focuses the light with an effective focal length, *EFL*, of

1 $$EFL=\frac{nD}{4(n-1)}$$

where *n* is the refractive index and *D* is the diameter of the sphere. This assumes that the sphere diameter is significantly larger than the wavelength of the incident light. As the focal point is close to the edge of the sphere, the focused light starts to diverge at a short distance from the sphere. This limits the maximum resist thickness that can be used without blanket exposing the photoresist. The minimum thickness of resist is constrained by practical fabrication limitations (e.g. the requirement for a protective etch layer).

In conventional photolithography, a mask is used to block the UV light in specific regions to provide feature contrast. The SA-MSL technique differs in that all of the incident light passes through the spheres but feature contrast is achieved by focusing the light to increase the exposure dose in particular areas. This makes the SA-MSL features much more sensitive to exposure dose.

### FDTD simulations

The finite-difference time-domain (FDTD)^16^ method is a computational approach which approximates and solves the two Maxwell’s curl equations on a fine three-dimensional grid. The maximum size of the space cells $\Delta x,\Delta y,\Delta z$ is constrained by the simulated time step Δ*t* for the algorithm to converge (Courant-Friedrich-Levy condition):

2 $$\Delta t < {{\textstyle (}c\sqrt{1/\Delta {x}^{2}+1/\Delta {y}^{2}+1/\Delta {z}^{2}}{\textstyle )}}^{-1}$$

where *c* is the speed of light in vacuum. This condition typically results in a large grid and a large number of time steps, which can be computationally intensive. However, the flexibility in simulating a variety of material properties, as well as the ability to visualise the propagation of broadband pulses, makes this method attractive for simulating optical and quasi-optical devices.

In this work, we have used custom FDTD software, Lucifer^17^, to study the light propagation throughout the microsphere array and photoresist, in order to identify situations where the contact areas between the spheres and the substrates produce interesting patterns, e.g. star shapes.

The simulation used a cubic cell with an edge of between 25 and 50 nm. Periodic boundary conditions were used on the sides of the simulation domain, while the top and bottom boundaries were absorbing. The light source has been modelled as a linearly polarised incoherent light source to account for the spectrum of the mercury lamp. A plane wave source is enforced by using periodic boundary conditions at each of the sides of the simulation domain. The spectral features measured from the mercury lamp of an EVG620 aligner, as used for the experiments (Fig. 1b), have been simulated using the Ornstein-Uhlenbeck process as defined in equation 3^18^.

3 $$X(t+\Delta t)=X(t){e}^{-(1/\tau )\Delta t}+{{\textstyle [}\frac{C\tau }{2}{\textstyle (}1-{e}^{-(2/\tau )\Delta t}{\textstyle )}{\textstyle ]}}^{\frac{1}{2}}n$$

where Δ*t* is the time step, *τ* is a constant which accounts for the line width, *C* is the diffusion constant and *n* is a pseudorandom number generated from a Gaussian distribution. The X value for each numerical time step is multiplied by a sine function at at the frequency of the lamp emission lines. Similarly Y is multiplied by a cosine. This is repeated for each of the peaks found in the spectrum and the *τ* values are optimised to correctly model the line widths.

The dielectric materials were modelled as perfect dielectrics, which included silicon, novolak based positive resist, and polystyrene microspheres with relative dielectric constants of 12^19^, 2.8^20^ and 2.5^21^ respectively.

The exposure of the resist has been calculated from integrating the light intensity (I) over time, where the light intensity was calculated by using the relationship in equation 4:

4 $$I=\frac{cn{\varepsilon }_{0}}{2}{|E|}^{2}$$

Each of the microspheres will be physically touching and are likely to be slightly deformed as the polystyrene is flexible. Therefore, in an hexagonally close-packed array, the spheres will have some small flat sections at the contact areas. The points of the stars emerged from the absence of reflective boundaries between adjacent spheres, essentially behaving as a uniform material with star-shaped focal points. This geometrical arrangement has been simulated by overlapping the spheres, which removes the reflective boundaries.

**Figure 2:**
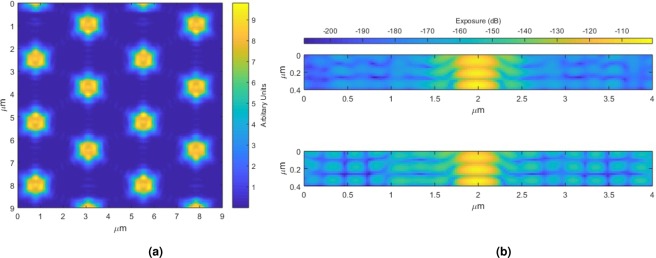
(**a**) Image showing the integrated light intensity through the resist below the 3 *μ*m spheres. (**b**) Images showing orthogonal cross sections through the resist below the 3 *μ*m spheres.

**Figure 3:**
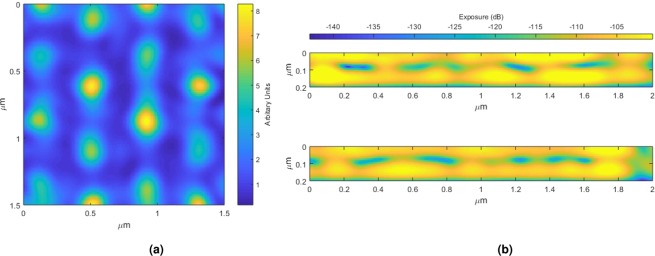
(**a**) Image showing the integrated light intensity through the resist below the 500 nm spheres. (**b**) Images showing orthogonal cross sections through the resist below the 500 nm spheres.

Figure 2b shows the integrated light intensity across the sections of the resist below the touching spheres. This can be seen in the comparison of the two images in Fig. 2b where the top image is perpendicular to the touching spheres and the bottom image is through the touching spheres. In general, in the photoresist surrounding the central axis of the sphere, the exposure is higher in the region along the star points (Fig. 2b bottom), whereas in the orthogonal direction, where there are no star points, the exposure is lower (Fig. 2b top). By exploiting this effect, it is possible to expose patterns with features which are significantly smaller than the spheres. Further reducing the sphere dimensions is challenging as it is more difficult to form an even monolayer of spheres^7^. Simulations have been repeated for various sphere dimensions, showing that the star pattern only appears for diameters above 1.5 *μ*m (≈3 wavelength). This has been confirmed experimentally using three different diameters of 3 *μ*m, 2 *μ*m and 500 nm. Simulations suggest that the star pattern is not present when there is strong diffraction from the spheres as shown in Fig. 3a,b, which smears out the smallest features. Therefore, if this method were to be repeated with a shorter wavelength, the minimum sphere size capable of producing the star shape would also be reduced.

**Figure 4:**
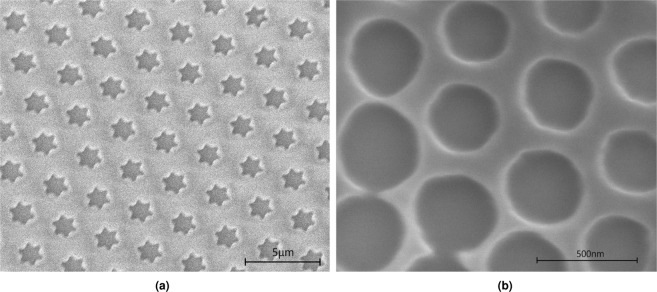
(**a**) SEM image at 5 kV of the feature array produced in uncoated 400 nm thick photoresist by a hexagonally close packed array of 3 *μ*m spheres with an applied exposure energy of 10 mJ/cm^2^. (**b**) SEM image at 4 kV of the feature array produced in uncoated 200 nm thick photoresist by a hexagonally close packed array of 500 nm spheres with an applied exposure energy of 10 mJ/cm^2^.

The experimental results in Fig. 4b which shows the circular holes formed by the 500 nm spheres, are consistent with the simulation results of Fig. 3a. As previously mentioned, these spheres are too small to produce a star pattern due to the increased diffraction. Similarly, Fig. 2a shows the simulation of exposure through the resist while 10 displays the experimental findings for the 3 *μ*m spheres. Here, the star pattern is clearly present and once again, the simulations are consistent with the experimental findings. Increasing the exposure level increases the size of the features, hence, although the pitch is directly proportional to the sphere size, there is some degree of control over the ratio of feature to pitch size.

Both theoretical and experimental findings conclude that the star shape is produced by removing the air-polystyrene reflective boundary between adjacent spheres, allowing homogeneous light transmission through the sphere monolayer. In Fig. 4a the points of the stars are directed towards neighbouring stars. The theoretical and experimental work shows that the star pattern is not observed when strong diffraction is present. The implication of these findings is that, with control of the microsphere arrangement, the feature shape in the photoresist can be controlled at the nanometre scale (square feature shapes have been found at lattice dislocations, see supplementary material). Multi-sized particle MSL could be an interesting avenue to explore here, possibly allowing feature shape tuning. This is significant since previous work have only reported simpler patterns, none of which were produced by only using a microsphere monolayer.

## Methods

SPR-350 positive photoresist was used on clean, 1 cm^2^ diced oxidised silicon <100> substrates to produce 0.4 *μ*m layers for the 3 *μ*m spheres and 0.2 *μ*m for the 2 *μ*m and 500 nm spheres. The 0.4 *μ*m thickness photoresist was deposited by mixing SPR-350 1.2 and EC solvent in a 2:1 ratio and spinning at a maximum speed of 7000 rpm. The 0.2 *μ*m thickness photoresist was deposited by mixing SPR-350 1.2 and EC solvent in a 1:2 ratio and spinning at a maximum speed of 3700 rpm.

The recipe detailed in^22^ was adapted and improved to have a larger uniformly-packed layer. The final recipe is shown in Table 1. There were some less populated areas and occasional lattice dislocations, however there is a consistent coverage of many ordered sections.

**Table 1:** Spin recipe used to self-assemble both the 2 *μ*m and 3 *μ*m microspheres.

Acceleration (R/s)	Speed (rpm)	Time (min:sec)
80	500	0:20
80	1000	1:30
100	2000	0:30
80	100	0:30

The microsphere formulation for both the 2 *μ*m and 3 *μ*m microspheres was 6% solids with 2% surfactant (Triton-X). The remainder of the solution was a mixture of water 60% and methanol 32%. Reference^23^ has been used as a guide for the 500 nm spin recipe and formulation using an acceleration of 100 R/s. This produced poor coverage: it was however sufficient to demonstrate the effects.

A mercury lamp in an EVG620 mask aligner in constant-energy mode was used to deliver a specified exposure to the top side of the sample, as characterised in Fig. 1b. The spectrum in this image shows the radiation as received by the sample through a glass plate. Each sample was developed in MF319 then rinsed in de-ionised water.

## Data Availability

Data for this paper is available at 10.15128/r10z708w44m.
